# Use of Protection for Unwanted Pregnancy and Sexually Transmitted Infections in Six Birth Cohorts in Norway 2020: A Descriptive Study

**DOI:** 10.1007/s12119-021-09879-w

**Published:** 2021-05-31

**Authors:** Bente Træen, Nantje Fischer

**Affiliations:** Department of Psychology, University of Oslo, Olso, Norway

**Keywords:** Contraception, Sexually transmitted infections, Unwanted pregnancy, Sexual intercourse, Condom use

## Abstract

This study describes the use of contraception and protection for sexually transmitted infections (STIs) in six different birth cohorts of the general population in Norway. The results are based on a 2020 national web panel survey among 18–89 year-olds in Norway (n = 4160). For respondents born within 1931–1950 versus those born within 1990–2002, there was a significant increase in the use of protection against unwanted pregnancy and STIs during sexual intercourse, and a significant drop in the proportion of those who did not use any protection at all. More women today (than in previous decades) are using hormonal contraception. The main reason for not using condoms during intercourse was both parties felt safe that they were healthy, especially those born within 1990–2002. To prevent unwanted pregnancy and STIs, it is beneficial to continue to increase the availability of free or subsidized hormonal contraception, including emergency contraception, and free condoms in public arenas that people frequent and where they meet their partners.

## Introduction

Sexual values, norms, and behaviour within a society or culture are characterized by stability rather than change (Simon & Gagnon, 1986). At the individual level, changes in patterns of sexual behaviour occur in the transition from one phase of life to another (Gagnon & Simon, 2005; Lewin, 1990, 1991; Simon & Gagnon, 1986). Society expects and tolerates different patterns of sexual behaviour from its members according to the phase of life they are in. From the rather limited experiences of sexuality in childhood, the teenager transits to a more comprehensive pattern of sexual behaviour and is, as a responsible citizen, expected to use contraception to avoid unwanted pregnancy (Gagnon & Simon, 2005; Lewin & Helmius, 1983). If pregnancy is not aimed for, married or cohabiting couples are expected to be monogamous, use contraception until menopause, and develop varied patterns of sexual behaviour within their relationship (Schmidt, 1989). People of fertile age use condoms for protection against unwanted pregnancy but often find it unnecessary to use condoms to protect against sexually transmitted infections (STIs; Træen & Hovland, 1998). They fear being regarded as suspicious and trust the partner would tell them if they were infected with STIs (Træen & Gravningen, 2011). After menopause, women are no longer fertile, and protection against unwanted pregnancy is no longer warranted. However, older adults continue to have sex with their partner(s) and engage in new relationships. This raises the question of STI protection, and there is evidence of an increased number of older adults contracting STIs (Schwartz et al., 2014). It is possible that older adults do not perceive themselves as being at risk of STIs or that erectile difficulties impair the use of condoms. However, this may also be a generational effect. The sexual socialization process every individual goes through is shaped by the historical period in which he or she is born into and lives in. For this reason, the era in which people were born, grew up, and started sexual relations must be considered. Post-World War 2 Norwegians had their coital debut at the age of 17–20 years (Stigum et al., 2010), ushering in periods that offered different opportunities for contraceptive use. In this regard, we provide a short presentation of the central features of the development in Post-World War 2 Norwegian society.

Changes in people’s sexual behaviour can occur simultaneously with changes in structural and economic conditions in society (Lewin, 1990). Important macro-sociological changes have taken place in Post-World War 2 Norway, and such changes may influence the availability and use of contraceptive measures and methods. In the 1950s, new technological interventions revolutionized the housewife’s work day, and “youth culture” emerged with rock’n roll music. Condoms were the means of contraception, along with more unsecure methods, such as interrupted intercourse, secure periods, and pessary. The pill was first developed in the United States in 1956 and was made available on the market from 1960 in the United States and from 1967 in Norway. During the 1960s and the 1970s in Norway, new social groups entered universities. Primarily, these groups constituted the sons of the working class and the daughters of the middle class. As a consequence of this social mobility, the percentage of the population belonging to the middle class grew (Ramsøy, 1977). Middle-class norms, values, attitudes, and behaviour were reflected in larger segments of the population. Furthermore, Norway experienced economic growth, as oil was found in the North Sea. These macrosocial changes also had consequences in the area of sexuality. Of great importance during this period was access to the pill, which freed some women from the fear of unwanted pregnancy. Furthermore, homosexuality was decriminalized in 1972 and removed as a psychiatric diagnosis in 1976. The happy-go-lucky sexual emancipation of the 60 s and 70 s was the background for a large gonorrhoea campaign at the beginning of the 70 s.

The central features of Norway during the 1980s were cultural urbanization, a philosophy of individualism, and fragmentation of public life and life in general (Hylland Eriksen, 1993). Those who grew up or were young during the 1980s may be regarded as products of the social and cultural process of individualization in Norwegian society (Hylland Eriksen, 1993), which is best characterized by terms such as flux, movement, negotiation, and risk (Beck, 1992; Beck et al., 1994; Crook et al., 1992; Featherstone, 1990; Giddens, 1991). Instead of ontological and epistemological certainty—albeit often of a constricting sort—people were subjected to all kinds of uncertainty and various types of risk. In the past decade, sexual emancipation was substituted in the 80 s and 90 s with the fear of viruses and a loss of innocence. In the wake of the human immunodeficiency virus (HIV) and acquired immunodeficiency syndrome (AIDS) epidemic in the 1980s, sex became dangerous. The authorities took on the responsibility of protecting the members of society from contracting the disease. Risk groups of the population for HIV and STIs have become important target groups for AIDS-preventive efforts. At the same time, the health authorities continued their work to prevent unwanted pregnancies and abortions. Condoms were particularly promoted for men who have sex with men (MSM) and adolescents. For heterosexual adolescents, condom use was promoted in addition to the use of oral contraception if pregnancy was not aimed for. Furthermore, the AIDS epidemic is likely to have had an impact on attitudes towards LGBT people (Ruel & Campbell, 2006). The Nordic countries are distigguished by an ideology about equality between individuals and groups of individuals. It is therefore not surprising that Kindeberg and Christensson (1994) within this cultural context found, in a longitudinal study among Swedish 15–18 years-olds, that the AIDS epidemic contributed to more positive attitudes towards LGBT people.

As a financial crisis occurred at the beginning of the 1990s, many Norwegians were thrown into economic chaos. At the same time, new types of hormonal contraception were developed and became available on the market. From the late 1990s to 2010, there was financial growth, and medication for HIV became available. This may be why the pressure on advocating for the use of STI protection, in addition to preventing unwanted pregnancy, diminished. This occurred despite the emergence of antibiotic-resistant microbes causing gonorrhoea and syphilis, particularly in subgroups of the population such as MSM.

From 2002, the availability of contraception and STI protection increased as part of preventive work. The Directorate of Health launched a program that gave condoms for free to adolescents and groups of the population at particular risk for STIs (e.g., MSM). The sales statistics for condoms increased from 400,000 in 1999 to approximately 2.5 million in 2007 (Træen & Gravningen, 2011). Emergency contraception was obtainable without prescription from a doctor. From 2002 to 2005, persons aged 15–19 years had access to free hormonal contraception from any doctor, or from a nurse or midwife working at public health clinics for adolescents. Since 2006, contraception has no longer been provided for free, but was subsidized for persons below 25 years of age. In 2007, 64,000 women aged 16–19 received a prescription for oral contraception, compared with 62,000 in 2006 (National Institute of Public Health, 2008). The effect of these preventive efforts on contraceptive behaviour seemed positive on the actual use (Øren et al., 2010). These changes occurred at the time of financial growth, but around 2010, another financial crisis occurred. In the 2020s, the Norwegian economy was sound. However, to date, little research has investigated how these macrosocial changes are reflected in people’s use of contraceptives and methods for STI protection.

### Purpose

This study described the use of contraception and STI protection among Norwegian men and women across six birth cohorts—(1) during their first ever sexual intercourse, (2) during their first sexual intercourse with their most recent sexual partner, and (3) across age groups during their most recent intercourse—to understand their behaviour and determine the reasons for not using condoms.

## Methods

### Participants

The study’s respondents were recruited between March and April in 2020 by e-mail from a randomly selected sample of 11,685 Norwegians registered in Kantar’s Gallup Panel. Kantar is the world's leading data, insights and consulting company. Of those 11,685 who were invited to participate, a total of 4160 individuals aged 18–89 years completed the survey, yielding a response rate of 35.6%. The participants were divided into six birth cohorts (528 individuals born 1931–1950, 560 born 1951–1960, 723 born 1961–1970, 623 born 1971–1980, 844 born 1981–1990, and 882 born 1991–2002). Nearly half of the participants (51%) completed the survey using a mobile device.

Approximately 46,000 members of the Gallup Panel were randomly recruited based on questionnaire surveys conducted by phone using probability samples. Thus, self-recruitment was not possible. Members of the Gallup Panel are representative of Norway’s Internet population, that is, 98% of the population with access to the Internet (see http://www.medienorge.uib.no/english/). The members come from different backgrounds in terms of age, gender, occupation, education, income, media habits, consumer habits, and matters related to politics, culture, sports, and so on. This allowed Kantar to draw specific target groups representing a desired sample.

The members of the Gallup Panel were guaranteed safety and anonymity, and participation in the surveys was voluntary. Kantar operated with a carefully planned incentive program. A small incentive was given to motivate participation, but it was not sufficiently large to be the cause of participation in these surveys. All relevant studies followed the ethical guidelines developed for market and poll organization surveys (https://www.tnsglobal.com/press-release/we-are-strongly-committed-ethical-and-sustainable-practices). A pilot survey was approved by the internal ethics committee of the Department of Psychology, University of Oslo.

### Survey Questions

The questionnaire used in the study was developed by a group of researchers at the Department of Psychology, University of Oslo, based on experience with the 2013 Norwegian Sex Study of 18–29 year-olds (Kvalem et al., 2014; Træen et al., 2016). The average time to complete the survey was 15 min. Prior to the survey, the questionnaire was piloted on a self-selected Facebook sample.

### Sociodemographic Characteristics of the Sample

Of the participants, 2181 (52.4%) were men, 1967 (47.3%) were women, and 12 participants indicated that they had another gender identity (0.3%). Based on Kantar’s data, three of the participants were classified as men and nine as women. The mean age of the participants was 46.5 years (SD 17.1 years), and the median age was 44.0 years (range 18–89 years). The mean age of the men was 48.4 years (SD 17.1 years); the median was 48.0 years (range 18–87 years). Meanwhile, the mean age of the women participants was 44.4 years (SD 16.8 years); the median was 41.0 years (range 18–89 years). The proportion of those who reported being heterosexual was 93.5%; 2.6% were homosexual/lesbian, 3.3% bisexual, and 0.6% asexual. Most participants reported that they had no religious affiliation (59.5%), and 38.7% were Christians, mainly Protestants or Christians with no particular denomination. The proportion of participants with a short university education (bachelor’s degree) was 41.4%, and 22.8% reported long university education (for instance, master’s degree or PhD). Regarding place of residence, the majority of participants lived in urban areas (56.8%), and only 16.3% lived in rural areas. Most participants reported living with a partner (63.4%); 25.4% reported being unmarried, 8.4% separated/divorced, and 2.8% widow/widower.

### Measures

*Contraceptive use* during sexual intercourse on different occasions was measured by three questions as follows:

1. Did you use contraception/protection the first time you had sexual intercourse?,
2. Did you use contraception/protection the first time you had intercourse with your most recent partner?, and
3. Did you use contraception/protection the most recent time you had intercourse with your partner?

The response categories to all three questions were: (1) No/none, (2) Condom, (3) Both condom and other contraception, (4) Hormonal contraception (oral contraceptives, rings, patches, syringes, hormone IUD), (5) IUD, (6) Pessary, (7) Spermicides, (8) Interrupted intercourse or safe periods, (9) Emergency contraception, (10) Sterilization, (11) Other protection, (12) Uncertain/Don’t know, and (13) Prefer not to answer.

*Reasons for non-use of condoms* were explored by asking:

1. Why did you not use condoms to protect yourself against STIs during your first intercourse (tick all that apply)?, and
2. Why did you not use a condom to protect yourself against STIs during your most recent intercourse with your partner (tick all that apply)? The response alternatives to both questions were: (1) Wanted to become pregnant, (2) I felt safe that we were both healthy, (3) Condom use would cause erectile difficulties, (4) Sex with condoms is less sensual, (5) Because of alcohol/drugs, (6) My partner did not want to use condoms, (7) There was no condom available, (8) I did not have money to buy condoms, (9) I think it’s embarrassing to buy condoms, (10) I was afraid my partner would think I’m changing sex partners often, (11) Other reasons, and (12) Prefer not to answer.

*Relationship with sexual partner* was measured by the question, “What was your relationship with your most recent partner?” The response categories were permanent partner, ex (previous) partner, friend, casual contact, prostitution contact, other, and prefer not to answer.

*The birth cohort* was assessed by year of birth and subsequently recoded into six categories: 1 = 1931–1950 (age group 70+ years), 2 = 1951–1960 (age group 60–69 years), 3 = 1961–1970 (age group 50–59 years), 4 = 1971–1980 (age group 40–49 years), 5 = 1981–1998 (age group 30–39 years), and 6 = 1999–2002 (age group 18–29 years).

### Statistical Methods

All data analyses were performed using SPSS 25.0 for Windows. A contingency table analysis was used to examine group differences.

## Results

### First Intercourse

Table 1 shows data on the use of contraception and STI protection during the first sexual intercourse of men and women across six different birth cohorts. Condoms and hormonal contraception are the two main methods of contraception used in Norway. Across all birth cohorts, 40–50% of the respondents reported using condoms during their first sexual intercourse. Among the oldest participants, it was also quite common to report interrupted intercourse or safe periods.

**Table 1:** Use of contraception/protection at the first occasion of sexual intercourse in Norwegian men and women, by birth cohort (percent)

	Men							Women						
Birth cohort	1931–1950	1951–1960	1961–1970	1971–1980	1981–1990	1991–2002	*p*	1931–1950	1951–1960	1961–1970	1971–1980	1981–1990	1991–2002	*p*
Age group	70+	60–69	50–59	40–49	30–39	18–29		70+	60–69	50–59	40–49	30–39	18–29	
None	49.5	47.5	47.3	39.9	20.8	17.4	.000	38.0	33.1	36.9	27.8	19.3	10.4	.000
Condom	40.7	41.1	38.5	39.3	46.5	43.4		44.3	50.6	43.2	53.8	49.7	47.3	
Condom and other contraception	0.7	1.1	2.3	3.1	8.8	12.8		–	0.8	3.8	4.0	11.7	18.2	
Hormonal contraception	2.0	3.4	5.6	8.8	15.0	20.7		2.5	7.8	9.4	8.3	11.5	18.0	
IUD	1.0	0.8	0.5	0.3	–	1.0		–	1.2	–	–	–	–	
Pessary	0.3	0.4	0.3	–	–	–		1.3	0.4	–	–	0.3	–	
Spermicides	0.3	–	0.3	–	–	0.3		–	–	–	0.4	–	–	
Interrupted intercourse or safe periods	4.1	1.9	1.8	2.5	1.8	0.3		10.8	3.9	1.4	1.8	1.0	1.1	
Emergency contraception	–	–	0.3	0.3	1.8	1.3		–	–	–	0.4	1.6	4.1	
Sterilization	–	–	–	–	0.3	–		–	–	–	–	–	–	
Other protection/Uncertain	1.4	3.8	3.3	5.7	5.3	2.6		2.8	2.3	5.2	3.6	4.9	0.9	
N =	295	265	395	318	400	304		158	257	287	277	384	444	

Tested for statistically significant group differences by means of Chi-square test (men: *χ*^2^_(55)_ = 318.565; women *χ*^2^_(50)_ = 365.754

**Fig. 1:**
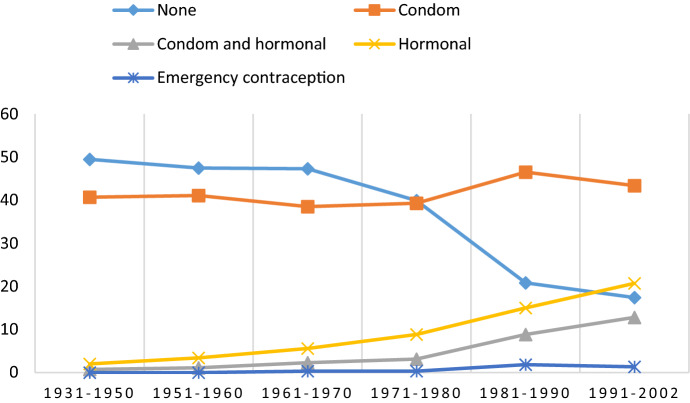
Use of contraception/protection during first intercourse by birth cohort in men (percent)

**Fig. 2:**
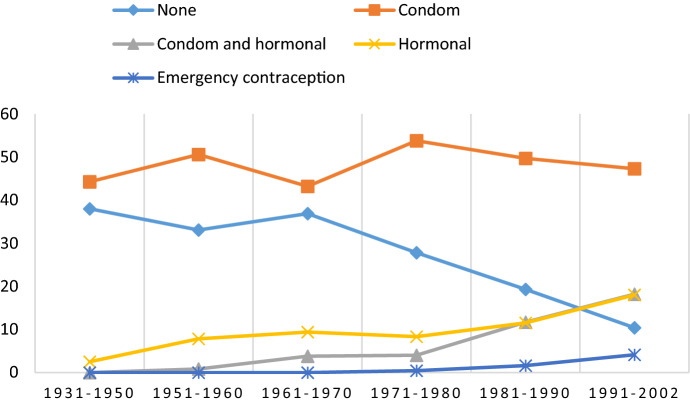
Use of contraception/protection during first intercourse by birth cohort in women (percent)

Some trends in reporting were observed (see Figs. 1, 2). First, the proportion of non-users of contraception during the first sexual intercourse dropped from 49.5% among men born within 1931–1950 to 17.4% among men born within 1991–2002, and the corresponding figures among women were 38.0% and 10.4%, respectively. Second, the proportion of respondents who reported using hormonal contraception increased from 2.0% among men born within 1931–1950 to 20.7% among men born within 1991–2002. The corresponding proportions among women were 2.5% and 18.0%, respectively. Third, an increasing trend in the number of people using both condoms and hormonal contraception during the first sexual intercourse was observed, with proportions of 12.8% for men and 18.2% for women born within 1991–2002.

Across all birth cohorts, the most reported motive for not using condoms during the first intercourse was that there was no condom available, even though the proportion of those who reported this declined when comparing the oldest to the youngest birth cohort in men (see Table 2). Another frequent motive among men and women was feeling safe that both parties were healthy, particularly those born within 1991–2002. With the exception of women born within 1991–2002, a relatively high proportion of women reported “other reasons” for not using condoms. Lastly, the proportion of those who reported the use of alcohol or drugs as a motive for not using condoms increased from 4.8% among men from the oldest birth cohort to 18.9% among men from the youngest birth cohort. The respective proportions among women were 3.5% and 24.0%, respectively.

**Table 2:** Reasons for not using condoms for STI protection during first intercourse in Norwegian men and women, by birth cohort (percent)

	Men							Women					
Birth cohort	1931–1950	1951–1960	1961–1970	1971–1980	1981–1990	1991–2002		1931–1950	1951–1960	1961–1970	1971–1980	1981–1990	1991–2002
Age group	70+	60–69	50–59	40–49	30–39	18–29		70+	60–69	50–59	40–49	30–39	18–29
I and/or my partner wanted to become pregnant	4.2	4.7	–	2.8	0.6	0.8		8.2	4.2	1.4	0.9	0.7	0.7
I felt safe we were both healthy	41.7	43.6	37.7	38.2	47.1	52.3		29.4	34.5	24.5	35.1	26.8	42.7
Condom use would cause erectile difficulties for me or my partner	1.8	1.3	1.3	2.2	3.4	6.8		–	0.8	–	–	0.7	–
Sex with condoms is less sensual	8.9	8.1	4.8	5.1	9.8	15.9		2.4	2.5	1.4	1.8	2.8	6.7
Because of alcohol/drugs	4.8	10.7	13.4	20.8	19.0	18.9		3.5	8.4	15.0	18.9	17.6	24.0
My partner did not want to use condoms	4.2	2.0	3.9	3.9	5.2	3.8		5.9	–	5.4	1.8	7.7	13.3
There was no condom available	45.2	36.9	42.4	38.2	30.5	31.1		28.2	34.5	32.0	37.8	34.5	36.7
I did not have money to buy condoms	1.8	0.7	1.3	1.7	2.3	0.8		–	0.8	1.4	1.8	–	–
I think it’s embarrassing to buy condoms	7.1	4.7	4.3	1.1	2.9	3.0		2.4	2.5	2.0	1.8	0.7	2.0
I was afraid my partner would think I’m changing sex partners often	–	–	–	0.6	0.6	–		–	0.8	0.7	–	–	0.7
Other reasons	8.9	5.4	9.5	11.8	14.4	15.9		27.1	21.8	29.3	16.2	25.4	10.0
N =	168	149	231	178	174	132		85	119	147	111	142	150

Percentages exceed 100%, because multiple responses were possible

### First Intercourse with Most Recent Partner

**Fig. 3:**
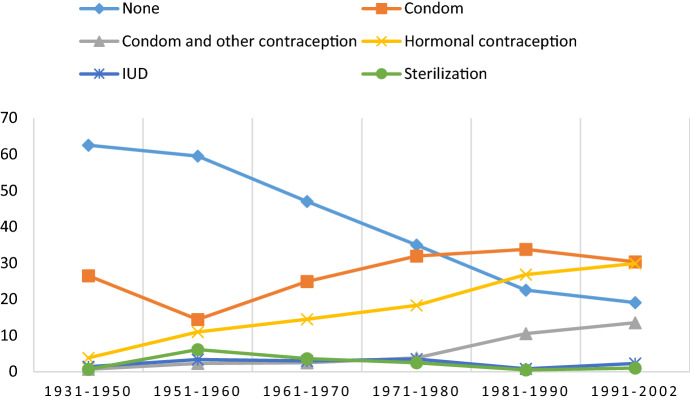
Use of contraception/protection at the *first* occasion of sexual intercourse with the *most recent* partner in men by birth cohort (percent)

**Fig. 4:**
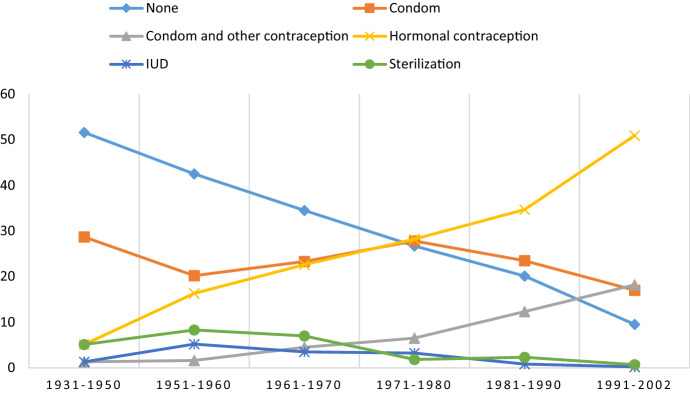
Use of contraception/protection at the *first* occasion of sexual intercourse with the *most recent* partner in women by birth cohort (percent)

The first sexual intercourse with a new partner poses a potential risk for STIs, and condoms should ideally be used for protection. Table 3 shows data on the use of contraception and STI protection during this event. The reporting of non-use of any means or methods of contraception/protection decreased substantially when comparing respondents from the oldest to the youngest birth cohorts among genders. Condom use was the most commonly reported type of contraception among men across birth cohorts. While condoms were the most frequently reported contraception among women born before 1960, for the youngest birth cohorts (1981–2002), hormonal contraception was the most common. The use of both hormonal contraception and condom and other contraception increased substantially when comparing the oldest and youngest birth cohorts. The main trends are visualized in Figs. 3 and 4.

**Table 3:** Use of contraception/protection at the *first* occasion of sexual intercourse with the *most recent* sex partner in Norwegian men and women, by birth cohort (percent)

	Men							Women						
Birth cohort	1931–1950	1951–1960	1961–1970	1971–1980	1981–1990	1991–2002	*p*	1931–1950	1951–1960	1961–1970	1971–1980	1981–1990	1991–2002	*p*
Age group	70+	60–69	50–59	40–49	30–39	18–29		70+	60–69	50–59	40–49	30–39	18–29	
None	62.5	59.5	47.0	35.0	22.5	19.1	.000	51.6	42.5	34.5	26.7	20.1	9.5	.000
Condom	26.5	14.4	24.9	31.9	33.8	30.3		28.7	20.2	23.3	27.8	23.5	17.0	
Condom and other contraception	0.7	2.3	2.5	3.8	10.5	13.5		1.3	1.6	4.5	6.5	12.3	18.2	
Hormonal contraception	3.8	11.0	14.5	18.3	26.8	29.9		5.1	16.3	22.6	28.2	34.7	50.9	
IUD	1.4	3.4	3.0	3.5	0.8	2.3		1.3	5.2	3.5	3.2	0.8	0.2	
Pessary	0.3	0.4	0.3	–	–	–		–	0.8	0.3	–	0.5	–	
Spermicides	0.3	–	0.3	–	–	–		–	–	–	0.4	–	–	
Interrupted intercourse or safe periods	1.4	1.5	1.3	1.9	1.5	0.7		3.2	2.0	1.0	2.5	1.6	0.7	
Emergency contraception	–	–	–	–	0.8	0.7		–	–	–	1.4	0.8	0.9	
Sterilization	0.7	6.1	3.6	2.5	0.5	1.0		5.1	8.3	7.0	1.8	2.3	–	
Other protection/Uncertain	2.4	1.5	2.8	3.2	3.1	1.0		3.8	3.2	3.1	1.4	3.4	2.5	
N =	291	264	394	317	400	304		157	252	287	277	383	440	

Tested for statistically significant group differences by means of Chi-square test (men: *χ*^2^_(55)_ = 396.913; women: *χ*^2^_(55)_ = 440.331)

### Most Recent Intercourse

**Fig. 5:**
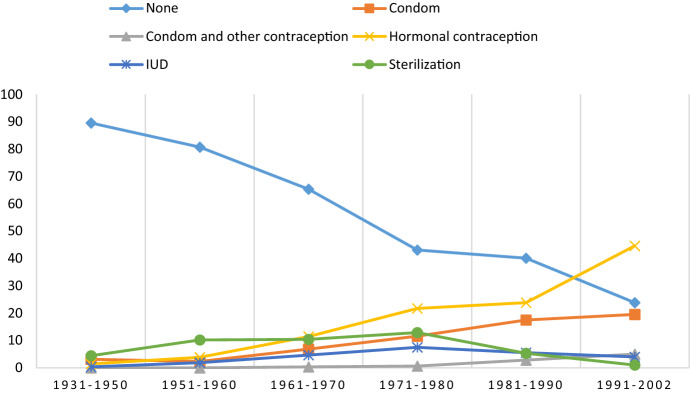
Use of contraception/protection at the *most recent* occasion of sexual intercourse in men by birth cohort (percent)

**Fig. 6:**
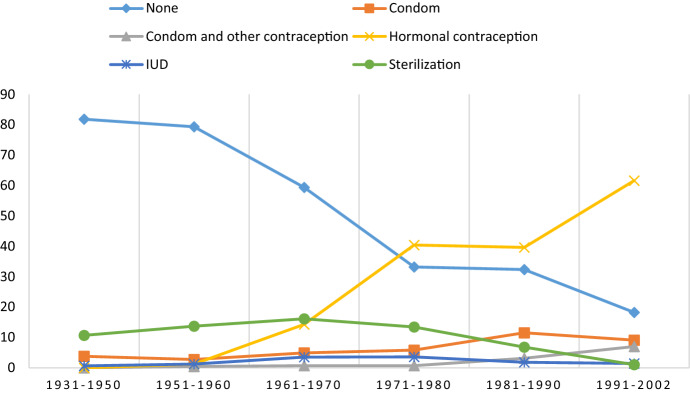
Use of contraception/protection at the *most recent* occasion of sexual intercourse in women by birth cohort (percent)

Table 4 shows the use of contraception and STI protection during the most recent sexual intercourse. Across gender and birth cohorts, the number of non-users of contraception decreased substantially when comparing respondents from the oldest to the youngest birth cohorts. However, hormonal contraception increased from 1.4% among men born within 1931–1950 to 44.6% among men born between and 1991–2002. None of the women in the oldest birth cohort, and only 1.2% among those born within 1951–1960, reported using hormonal contraception, but there was an increase of up to 61.6% in the use among women born within 1991–2002. Condom use was reported by 3.1% of men born within 1931–1950, and this figure increased to 19.5% for men born within 1991–2002. Among men and women born prior to 1980 (aged 40+ years), 10–15% reported sterilization. The other contraceptive measures and methods have not been widely reported by either gender. The observed trends are visualized in Figs. 5 and 6.

**Table 4:** Use of contraception/protection at the *most recent* occasion of sexual intercourse in Norwegian men and women, by birth cohort (percent)

	Men							Women						
Birth cohort	1931–1950	1951–1960	1961–1970	1971–1980	1981–1990	1991–2002	*p*	1931–1950	1951–1960	1961–1970	1971–1980	1981–1990	1991–2002	*p*
Age group	70+	60–69	50–59	40–49	30–39	18–29		70+	60–69	50–59	40–49	30–39	18–29	
None	89.5	80.7	65.3	43.1	40.1	23.8	.000	81.8	79.3	59.4	33.2	32.3	18.2	.000
Condom	3.1	2.3	6.8	11.6	17.5	19.5		3.8	2.7	4.9	5.8	11.5	9.1	
Condom and other contraception	–	–	0.3	0.6	2.8	5.0		–	0.4	0.7	0.7	3.1	7.0	
Hormonal contraception	1.4	3.8	11.4	21.7	23.8	44.6		–	1.2	14.3	40.4	39.6	61.6	
IUD	0.3	1.9	4.6	7.5	5.5	4.0		0.6	1.2	3.5	3.6	1.8	1.4	
Pessary	–	–	–	–	–	–		–	–	–	–	–	–	
Spermicides	–	–	–	–	–	–		–	–	–	–	–	–	
Interrupted intercourse or safe periods	0.3	0.4	0.5	1.3	2.5	0.7		–	–	0.3	2.2	2.9	0.9	
Emergency contraception	–	–	–	–	–	–		–	–	–	–	–	0.5	
Sterilization	4.4	10.2	10.4	12.9	5.3	1.0		10.7	13.7	16.1	13.4	6.8	0.7	
Other protection/Uncertain	1.0	0.8	0.8	1.2	2.6	1.6		3.1	1.6	0.6	0.8	1.8	0.7	
N =	294	264	395	318	399	303		159	256	286	277	381	440	

Tested for statistically significant group differences by means of Chi-square test (men: *χ*^2^_(40)_ = 605.604; women: *χ*^2^_(45)_ = 696.194)

Across all birth cohorts, the most reported motive for not using condoms during their most recent intercourse was that both partners felt safe that both were healthy (see Table 5). This motive was reported by 71–81% of men and 57–75% of women. In addition, a relatively high proportion of women and men reported “other reasons” for not using condoms. The proportion of those reporting “sex with condoms is less sensual” increased from 7.2% among men from the oldest birth cohort to 27.3% in the youngest birth cohort. The proportions among women ranged from 0 to 15.5%.

**Table 5:** Reasons for not using condoms for STI protection during the *most recent* intercourse in Norwegian men and women, by birth cohort (percent)

	Men							Women					
Birth cohort	1931–1950	1951–1960	1961–1970	1971–1980	1981–1990	1991–2002		1931–1950	1951–1960	1961–1970	1971–1980	1981–1990	1991–2002
Age group	70+	60–69	50–59	40–49	30–39	18–29		70+	60–69	50–59	40–49	30–39	18–29
I and/or my partner wanted to become pregnant	0.4	0.8	1.4	5.2	13.4	5.3		0.7	0.4	1.6	2.4	10.3	7.9
I felt safe we were both healthy	71.8	70.6	81.3	77.9	73.9	77.1		57.3	66.7	68.6	75.0	74.8	72.5
Condom use would cause erectile difficulties for me or my partner	2.9	1.6	1.7	3.0	3.2	9.3		1.4	0.4	0.4	1.2	2.5	3.0
Sex with condoms is less sensual	7.2	7.9	7.2	11.2	16.9	27.3		–	1.7	1.9	2.0	9.0	15.5
Because of alcohol/drugs	–	–	1.1	0.7	2.5	2.6		–	0.4	0.8	2.0	1.6	6.0
My partner did not want to use condoms	3.2	4.4	2.5	3.7	4.5	7.0		–	–	0.4	2.0	2.8	5.4
There was no condom available	2.5	2.0	2.2	4.5	5.1	4.4		0.7	0.8	1.9	3.2	4.0	6.5
I did not have money to buy condoms	–	–	–	–	–	0.9		–	–	–	–	–	0.3
I think it’s embarrassing to buy condoms	0.4	–	–	–	0.3	1.3		–	–	–	–	–	–
I was afraid my partner would think I’m changing sex partners often	–	0.8	–	–	–	–		–	–	–	–	0.6	–
Other reasons	29.2	24.6	17.1	17.2	14.6	14.1		42.0	36.7	31.4	21.0	15.3	16.1
N =	277	252	363	267	314	227		143	240	258	252	321	367

Percentages exceed 100%, because multiple responses were possible

The use of contraception/protection at the most recent intercourse should be understood in light of the sex partner. In all birth cohorts, it was most common to have had the most recent intercourse with a permanent partner (71–87%, see Table 6). In addition, more men and women have had their most recent intercourse with a person they know (ex/previous partner or friend) compared with casual contact or transactional sex. It was most common for respondents aged 18–29 years (born 1991–2002) to have had the most recent intercourse with a non-permanent partner (approximately 30%).

**Table 6:** Relationship with the most recent sex partner in Norwegian men and women, by birth cohort (percent)

	Men							Women						
Birth cohort	1931–1950	1951–1960	1961–1970	1971–1980	1981–1990	1991–2002	*p*	1931–1950	1951–1960	1961–1970	1971–1980	1981–1990	1991–2002	*p*
Age group	70+	60–69	50–59	40–49	30–39	18–29		70+	60–69	50–59	40–49	30–39	18–29	
Permanent partner	85.6	80.2	78.5	75.9	76.1	71.1	.000	81.1	85.7	86.5	80.2	83.5	70.8	.001
Ex (previous) partner	1.4	5.0	3.5	4.8	2.5	5.0		4.4	4.3	2.8	5.8	4.2	6.6	
Friend	10.6	10.3	11.4	8.3	11.1	11.0		10.7	6.6	8.3	9.7	8.6	13.4	
Casual contact/ someone I did not know in advance	1.4	3.1	5.3	7.0	7.0	10.3		1.3	2.3	1.4	3.2	3.1	7.3	
Prostitution contact	1.0	–	0.5	2.5	1.8	–		–	–	–	–	–	0.2	
Other	–	1.5	0.8	1.6	1.5	2.7		1.9	1.2	1.0	1.1	0.5	1.6	
N =	292	262	396	315	398	301		159	258	289	278	382	439	

Tested for statistically significant group differences by means of Chi-square test (men: *χ*^2^_(25)_ = 66.995; women: *χ*^2^_(25)_ = 54.495)

## Discussion

We set out to explore the development of contraception/protection use across six birth cohorts in post-World War 2 Norway. Some interesting trends in the use of contraception and STI protection have emerged. First, from the oldest to the youngest birth cohort, there was a significant increase in the use of protection against unwanted pregnancy and STIs during the first sexual intercourse, and a significant drop in the proportion of those who did not use any protection at all. Second, there are more women who use hormonal contraception today than in previous decades. Third, the main reason reported for not using condoms during intercourse was that both parties felt safe that they were healthy, which was especially true for the youngest birth cohorts.

The drop in the number of people who do not use any kind of contraception or protection during the first sexual intercourse is promising, as those who use contraception/protection at their first intercourse are shown to continue doing so in the future (Træen et al., 2011). We found that condoms are still the most widely used means of contraception/STI protection during the first sexual intercourse among young Norwegians. In a previous web panel study of 18–24 Norwegians (born 1989–1992), about 60% reported using condoms during their first intercourse (Træen et al., 2011). Compared with the previous study, the proportion of condom and hormonal contraception users during the first intercourse in the youngest birth cohort has increased. This development in user patterns of contraception/protection is likely attributable to health authorities’ efforts to make hormonal contraception and condoms more available to young people, as well as to better sexual health education in schools.

While condoms were most frequently used during the first intercourse in all birth cohorts, the use of hormonal conception is more common during the most recent sexual intercourse (except among those 60+ years). This finding is in line with previous studies showing a pattern of change in contraceptive behaviour, where those who use condoms or other types of contraception during their first sexual intercourse tend to switch to hormonal contraception when they have been coitally active for some time (Træen et al., 2002, 2011). Few of the oldest respondents reported the use of hormonal contraception during their most recent intercourse, and this is most likely due to having reached menopause or having intercourse with a permanent partner who is no longer fertile. The tendency to use contraception during the most recent intercourse to prevent unintended pregnancy and not STIs also seems to be reflected in a greater use of sterilization as a more permanent solution for anti-conception among the older birth cohorts.

Overall, in all areas studied (*first* intercourse, *first* intercourse *with the most recent partner*, and *most recent* intercourse) there has been an increase in the use of hormonal contraception when comparing the oldest to the youngest birth cohorts. We assume that this is most likely due to better availability, the cost of hormonal contraception, and the development of new and more user-friendly types of hormonal contraception in Norway. Although the increased percentage of women who use hormonal contraception is good for preventing unwanted pregnancies, hormonal contraception does not protect against STIs. Thus, hormonal contraception seems to function as a “sleeping pillow” for STI protection in later potentially risky situations for STIs.

Among those who are fertile, the use of contraception/protection during the most recent sexual intercourse should be understood in light of who the sex partner was (Bauman & Berman, 2005). Most respondents across birth cohorts had their most recent intercourse with a permanent partner, and it was more common to have had this intercourse with a person they knew (ex-partner or friend), compared with a casual partner or sex worker. This means that the majority of our respondents had their most recent intercourse with someone they were likely to feel socially and psychologically secure. In this context, it is reasonable to assume that their concern was protecting themselves against unwanted pregnancy and not against STIs during sexual intercourse. This assumption confirms previous conclusions regarding the use of condoms in Norway (Træen & Gravningen, 2011; Træen & Hovland, 1998; Træen et al., 2003). Until the age of menopause, people “naturally” assume that they are fecund, and if pregnancy is not aimed for, they are expected to use contraception (Træen, 2008). This is perceived as “normal” and “natural.” However, sexually transmitted infections are stigmatized and perceived as “dirty” (Newton & McCabe, 2008). Qualitative studies show that sexual partners who are known and familiar are perceived as trustworthy and safe (Hammer et al., 1996; Skidmore & Hayter, 2000). Therefore, individuals perceive it as rather “unnatural” that they or their sexual partners might be carriers of a sexual disease. This psychological mechanism—not feeling at risk because you know/trust your sex partner—is also reflected in reasons for not using condoms during the first intercourse and the most recent intercourse. Across birth cohorts, the majority of women (57–75%) and men (71–81%) reported that they did not use condoms during the most recent sexual intercourse because they felt safe that both partners were healthy. During the first intercourse, the non-use of condoms was attributed to not having a condom available and feeling safe that both parties were healthy, particularly among the youngest respondents. These findings highlight the paradoxical relationship between emotional affection/trust and effective STI protection, and correspond with previous studies, where mutual trust and fear of suspicion were the underlying motives for not using condoms during the first intercourse with the most recent partner (Træen & Gravningen, 2011). To base one's behaviour in a sexual situation on the assumption that it is “natural” to take precautions against STIs would most likely be received by the partner as an instance of being “unnaturally”’ suspicious.

The results from this study indicate certain recommendations for educational interventions by cohort. First, information campaigns to raise the awareness for STIs in the general population should be continued, and increased efforts should be used to reach the older segments of the population with the message that they are not immune to such infections. Second, to increase the availability of condoms for all age groups, and of hormonal contraception to individuals in fertile ages, are likely to be effective prevention strategies.

### Limitations

This study has some limitations. First, as the study was conducted during the COVID-19 lockdown in March–April 2020 in Norway, there is reason to believe that some people may have been less receptive and interested in participating in a study on sexual behaviour. It is not known how this non-response bias may have affected our findings. However, a previous supplementary study—determining whether respondents and non-respondents in the Norwegian sexual behaviour study showed different patterns of sexual behaviour—concluded that non-response was not associated with differences in sexual behaviour (Stigum, 1997). Other Nordic studies also indicate that non-response is fairly random with respect to sexual behaviour (Haavio-Mannila & Kontula, 2003; Kontula & Haavio-Mannila, 1995; Lewin et al., 2000). Although it cannot be ruled out that the non-respondents have different patterns of contraceptive behaviour from the respondents, we believe that non-participation is random rather than systematic. However, the majority of our respondents had a high level of education. According to official 2018 statistics by the Central Bureau of Statistics, 34.1% of the Norwegian population aged 16 years or older had a high level of education. In our opinion, this indicates that our sample is slightly, although not severely.

Second, the analysis in this study was based on cross-sectional data. Therefore, the statistical effects of birth cohort and age could not be separated. Third, there may be a memory bias present in the data on the first intercourse, as this event may be many years back in time, especially for the oldest birth cohorts. Finally, it is reasonable to assume that a social desirability bias may be present in the reporting of contraceptive behaviour.

## Conclusion

The most promising finding from this study is that people, to an increasing degree, use both condoms and hormonal contraception during their coital debut, and later in life, during the first sexual intercourse with a new partner. This implies that people’s perception of what is “normal” and “natural” conduct in sexual contexts may have changed slightly in the past one to two decades. However, for the majority of the population, sexual health educators are still attempting to change the perception of what is “normal” and “natural” in life in condom campaigns aimed at preventing STIs. Regarding the use of measures to prevent unwanted pregnancy, there are clear indications that increasing the availability of such measures also increases the actual use (Øren et al., 2010; Træen & Gravningen, 2011). The implications of this study are that to prevent unwanted pregnancy and STIs, it is beneficial to continue to increase the availability of free or subsidized hormonal contraception, including emergency contraception, and free condoms in public arenas that people frequent and where they meet partners. The presence of condoms in these arenas will also signal to people that STI is an issue to consider even in romantic contexts, thereby increasing the probability of actual condom use.

## References

[CR1] Bauman LJ, Berman R (2005). Adolescent relationships and condom use: Trust, love and commitment. AIDS and Behavior.

[CR2] Beck U (1992). Risk society: Towards a new modernity.

[CR3] Beck U, Giddens A, Lash S (1994). Reflexive modernization: Politics, tradition and aesthetics in modern social order.

[CR4] Crook S, Pakulski J, Waters M (1992). Postmodernization: Change in an advanced society.

[CR5] Featherstone M (1990). Global culture: Nationalism, globalization and modernity. A theory, culture and society special issue.

[CR6] Gagnon JH, Simon W (2005). Sexual conduct: The social sources of human sexuality.

[CR7] Giddens A (1991). Modernity and self-identity.

[CR8] Haavio-Mannila E, Kontula O (2003). Single and double sexual standards in Finland, Estonia, and St. Petersburg. Journal of Sex Research.

[CR9] Hammer JC, Fisher JD, Fitzgerald P, Fisher WA (1996). When two heads aren’t better than one: AIDS risk behaviour in college-aged couples. Journal of Applied Psychology.

[CR10] Hylland Eriksen T, Hylland Eriksen T (1993). Norway went crazy: Åttitallets stille kulturrevolusjon (Norway went crazy. The silent cultural revolution in the 1980s, in Norwegian). Typisk norsk Essays om norsk kultur (Typically Norwegian. Essays about Norwegian culture, in Norwegian).

[CR11] Kindeberg T, Christensson B (1994). Changing Swedish students' attitudes in relation to the AIDS epidemic. Health Education Research.

[CR12] Kontula O, Haavio-Mannila E (1995). Sexual pleasures: Enhancement of sex life in Finland, 1971–1992.

[CR13] Kvalem IL, Træen B, Lewin B, Stulhofer A (2014). Self-perceived effects of internet pornography use, genital appearance satisfaction, and sexual self-esteem among young Scandinavian adults. Cyberpsychology: Journal of Psychosocial Research on Cyberspace.

[CR15] Lewin B (1990). Potentials for change. Scandinavian Journal of Infectious Diseases.

[CR16] Lewin B (1991). Att omplantera sexualiteten. Om latin-amerikanska ungdomars sexuella socialition i Sverige [Replanting sexuality. The sexual socialization in Sweden among Latin-American adolescents, Swedish text].

[CR17] Lewin B, Fugl-Meyer K, Helmius G, Lalos A, Månsson SA (2000). Sex in Sweden: On the sex-life in Sweden 1996.

[CR18] Lewin B, Helmius G (1983). Ungdom och sexualitet; En sociologisk studie av ungdoms sexuella föreställingar och erfarenheter [Youth and sexuality: A sociological study of young people's sexual conceptions and experiences].

[CR19] Newton DC, McCabe MP (2008). Sexually transmitted infections: Impact on individuals and their relationships. Journal of Health Psychology.

[CR20] Øren, A., Leistad, L., & Haugan, T. (2010). Endres prevensjonsvaner og abortrate hos kvinner 20–24 år ved tilbud om gratis hormonell prevensjon? [Does contraceptive habits and abortion rate change in 20–24 year-old women being offered free hormonal contraception]? SINTEF. http://hdl.handle.net/11250/2467251.

[CR21] Ramsøy NR (1977). ‘Sosial mobilitet i Norge’ [Social mobility in Norway].

[CR22] Ruel E, Campbell RT (2006). Homophobia and HIV/AIDS: Attitude change in the face of an epidemic. Social Forces.

[CR23] Schmidt G (1989). Sexual permissiveness in Western societies. Roots and course of development. Nordisk Sexologi.

[CR24] Schwartz P, Diefendorf S, McGlynn-Wright A, Tolman DL, Diamond LM (2014). Sexuality in aging. APA handbook of sexuality and psychology.

[CR25] Simon W, Gagnon JH (1986). Sexual scripts: Performance and change. Archives of Sexual Behaviour.

[CR26] Skidmore D, Hayter E (2000). Risk and sex: Egocentricity and sexual behaviour in young adults. Health, Risk & Society.

[CR27] Stigum, H. (1997). Mathematical models for the spread of sexually transmitted diseases using sexual behaviour data. *Norwegian Journal of Epidemiology, 7*(suppl no. 5).

[CR28] Stigum H, Samuelsen SO, Træen B (2010). Analysis of coital debut in Norway. Archives of Sexual Behavior.

[CR29] The National Institute of Public Health (2008). Norwegian prescription database 2004–2007. Oslo: The National Institute of Public Health. Retrieved from the Internet. April 20.

[CR30] Træen B, Træen B, Lewin B (2008). Sexual health promotion. Sexology in context.

[CR31] Træen B, Gravningen K (2011). The use of protection for sexually transmitted infections (STIs) and unwanted pregnancy among Norwegian heterosexual young adults 2009. Sexuality & Culture.

[CR32] Træen B, Hovland A (1998). Games people play: Sex, alcohol and condom use among urban Norwegians. Contemporary Drug Problems.

[CR33] Træen B, Samuelsen SO, Roen K (2016). Sexual debut ages in heterosexual, lesbian, gay, and bisexual young adults in Norway. Sexuality & Culture.

[CR34] Træen B, Stigum H, Eskild E (2002). Contraception and STD protection among urban Norwegians. Culture, Health and Sexuality.

[CR35] Træen B, Stigum H, Hassoun J, Zantedeschi E (2003). Presexual alcohol consumption and use of condoms: A cross-cultural European study. Culture, Health and Sexuality.

[CR36] Træen B, Štulhofer A, Landripet I (2011). Young and sexual in Norway and Croatia: Revisiting the Scandinavian vs. Mediterranean gendered pattern of sexual initiation. International Journal of Sexual Health.

